# ACEMg-mediated hearing preservation in cochlear implant patients receiving different electrode lengths (PROHEARING): study protocol for a randomized controlled trial

**DOI:** 10.1186/s13063-016-1526-7

**Published:** 2016-08-08

**Authors:** Verena Scheper, Melanie Leifholz, Heiko von der Leyen, Miriam Keller, Ute Denkena, Armin Koch, Annika Karch, Josef Miller, Thomas Lenarz

**Affiliations:** 1 Department of Otolaryngology, Hannover Medical School, Carl-Neuberg-Str. 1, 30625 Hannover, Germany; 2 Cluster of Excellence Hearing4all, Hannover and Oldenburg, Germany; 3 Hannover Clinical Trial Center, Carl-Neuberg-Str. 1, 30625 Hannover, Germany; 4 Institute for Biostatistics, Hannover Medical School, Carl-Neuberg-Str. 1, 30625 Hannover, Germany; 5 Kresge Hearing Research Institute, University of Michigan, 4605 Medical Science Unit II, Ann Arbor, MI 48109-5616 USA

**Keywords:** Cochlear implant, Antioxidants, Free radicals, Hearing preservation, Residual hearing, EAS, Electric acoustic stimulation

## Abstract

**Background:**

The indications for a cochlear implant (CI) have been extended to include patients with some residual hearing. Shorter and thinner atraumatic electrodes have been designed to preserve the residual hearing in the implanted ear. However, the insertion of the electrode array into the cochlea, with potential mechanical trauma and the presence of this foreign body inside the cochlea, may lead to free radical formation and reduced blood perfusion of the cochlea which can result in the loss of residual hearing.

**Methods/design:**

In this single-center, randomized, placebo-controlled, double-blind phase II clinical trial the effect of free radical scavengers and a vasodilator on the residual hearing of 140 CI patients will be evaluated. The formulation is composed of β-carotene (vitamin A), ascorbic acid (vitamin C), dl-α-tocopherol acetate (vitamin E) and the vasodilator magnesium (Mg), or ACEMg. Medication is administered twice daily per os for approximately 3 months. The primary measure is based upon the reduction in postoperative low-frequency air-conducted pure-tone thresholds compared to preoperative thresholds in ACEMg-treated patients compared to those of a placebo group. Additionally, the effect of different electrode lengths (20, 24 and 28 mm) is analyzed. Study visits are scheduled 2 days before surgery, at first fitting, which is the adjustment and start of stimulation via CI 4 weeks after surgery and 3, 6, 9 and 12 months after first fitting. The primary endpoint is the air-conduction hearing loss at 500 Hz 3 months after first fitting. Additionally, speech recognition tests, hearing aid benefit in the implanted ear and electrophysiological measurements of implant function are assessed.

Since this is a blinded clinical trial and recruitment is still ongoing, data continue to accrue and we cannot yet analyze the outcome of the ACEMg treatment.

**Discussion:**

There is an unfulfilled need for new strategies to preserve acoustic hearing in CI patients. This study will provide first-in-man data on ACEMg-mediated protection of residual hearing in CI patients. Performing all surgeries and patient follow-up at one study site improves consistency in diagnosis and therapy and less variability in surgery, audiological test techniques and fitting. This approach will allow investigation of the influence of ACEMg on residual hearing in CI patients.

**Trial registration:**

The German Bundesinstitut für Arzneimittel und Medizinprodukte (BfArM) application number 4039192, was registered on 6 December 2013 with protocol amendment version 3.0 from 19 August 2014. EudraCT number: 2012-005002-22.

## Background

The cochlear implant (CI) is the standard treatment for unilateral and bilateral severe/profound hearing loss, both in adults and children. The CI stimulates the auditory nerve electrically through electrodes placed inside the inner ear, the cochlea. Recently, as speech understanding with the CI has improved, indication criteria have been extended towards patients with more residual hearing. At the same time, thinner and shorter atraumatic electrodes have been designed to preserve the (low-frequency) residual hearing in the implanted ear [1, 2]. If hearing has been preserved, then the electrical stimulation of the implant may be combined with a hearing aid in the same ear (electric acoustic stimulation, EAS).

The insertion of the electrode array into the cochlea with potential mechanical trauma, and the chronic presence of this foreign body inside the cochlea may induce loss of residual hearing. As Talbot and colleagues stated in 2004, a substantial acoustic hearing loss occurs in 24 % of EAS CI patients, and among them 13 % show a total loss of residual hearing [3]. More recent studies prove that the occurring problem is not yet solved since a large number of patients continue to suffer from partial or total loss of residual hearing. In 15 % of patients analyzed by Skarzynski a partial to total hearing loss was observed [4]. Other studies published a complete loss of residual hearing in 22.7 % of subjects and a partial loss of hearing in 50.1 % of the total patient population (*n* = 22) [5] or a mean threshold shift >15 dB 24 months after first fitting (FF) in 41.2 % (Cochlear Implant Model Hybrid-L24, *n* = 51) and 71.4 % (Cochlear Implant Model CI422, *n* = 28), respectively [6].

Experimental studies on the auditory system have demonstrated that antioxidants plus a vasodilator reduce both noise- and drug- (aminoglycoside)-induced inner ear pathology and hearing loss by over 75 % [7]. This formulation is β-carotene (converted in the body to vitamin A), ascorbic acid (vitamin C), dl-α-tocopherol acetate (vitamin E) and the vasodilator magnesium. Together (ACEMg) they are remarkably effective in vivo. Data indicate that free radicals play a key role in sensory and neural cell death and loss of residual hearing with CI implantation [8, 9] which reduces the benefits of implantation. The goal of ACEMg treatment is to eliminate one important contributing factor, the stress of surgery and implantation of a foreign body that may reduce residual acoustic hearing in the implanted patient.

Our PROHEARING clinical trial (PROtect residual HEARING) is the first human trial to assess the efficacy of ACEMg to prevent permanent hearing loss in CI patients with residual hearing.

## Methods/design

The PROHEARING trial is conducted in accordance with the Declaration of Helsinki and complies with the principles of Good Clinical Practice (GCP) and Good Manufacturing Practice (GMP). The protocol is approved by the Independent Ethics Committee (IEC) of Hannover Medical School (MHH) and by the national legal authority, the Bundesinstitut für Arzneimittel und Medizinprodukte (BfArM).

The trial is registered on the European Clinical Trials Database (EudraCT Number: 2012-005002-22). It is funded by a grant from the European Commission 7th Framework Program in Public Health research. The trial is being coordinated by the Clinic of Otorhinolaryngology of MHH (sponsor). The MHH Institutes for Biostatistics and for Clinical Pharmacology support the trial with their expertise and the Hannover Clinical Trial Center (HCTC) is the management center of the study and responsible for clinical monitoring and data management. The trial complies with the principles of GCP and is carried out in accordance with applicable legislation and the Standard Operating Procedures of MHH.

The PROHEARING trial is a single-center, randomized, placebo-controlled, double-blind, phase II trial including adults admitted to the MHH Department of Otorhinolaryngology who are suffering from hearing loss, have a defined level of residual hearing and receiving a cochlea implant.

The study has the primary objective to demonstrate that ACEMg is more efficacious than placebo in preserving residual hearing during cochlear implantation by comparing the hearing loss 3 months after FF at 500 Hz in air-conducted pure-tone audiometry.

Key secondary objectives are:

- To investigate the drug effect over time (hearing loss in ACEMg compared to placebo at 500 Hz 6, 9 and 12 months after FF)
- To compare ACEMg and placebo for hearing loss at different frequencies of pure-tone audiometry (125, 250 and 750 Hz, 1, 1.5, 2, 3, 4, 6 and 8 kHz) over time (months 3, 6, 9 and 12 after FF)
- To compare efficacy by means of speech perception, functional hearing and impedances
- To evaluate the effect of electrode length on hearing loss

### Inclusion and exclusion criteria

The patient must meet specific criteria during consultation with the ENT surgeon before inclusion and randomization. The appropriate examinations will be performed to make sure that the patients meet the exclusion and inclusion criteria. During the study, the patient has the right to leave the study at any time. If the patient decides to stop the intake of the study medication he may continue the study and will be further followed up.

Patients will be eligible for the trial if they fulfill all the inclusion criteria (Table 1) and none of the exclusion criteria (Table 2).

**Table 1:** Inclusion criteria of the PROHEARING clinical trial

	Inclusion criteria
1.	18 years of age or older
2.	No or little benefit from a conventional hearing aid, defined as preoperative auditory speech understanding of less or equal 60 % in Freiburger monosyllables at 65 dB SPL, best aided in the ear to be implanted
3.	Residual hearing better or equal than 85 dB HL at 125, 90 dB HL at 250 Hz and better or equal than 95 dB HL at 500 Hz in the ear to be implanted
4.	Ability to understand the study procedures, possible risks and benefits, and to give informed consent
5.	Informed Consent Document is signed.
6.	Patients must agree not to use daily vitamin preparations containing vitamins A, C or E or magnesium during the course of the study, and beginning at least 48 hours prior to first intake of the study medication
7.	Female patients 50 years of age or older at the day of inclusion who have been postmenopausal for at least 1 year *Or* Female patients who have a negative hCG serum pregnancy test and meet one or more of the following criteria: are 6 weeks after surgical sterilization by bilateral tubal ligation or bilateral ovariectomy with or without hysterectomy are using proven oral, injected or implanted hormonal contraceptive methods: intrauterine device or intrauterine system barrier methods: condom or occlusive cap (diaphragm or cervical/vault caps) *with* spermicide (foam/gel/film/cream/suppository) male sterilization (if the absence of sperm in the ejaculate is documented. For female participants the vasectomized male partner should be the sole sexual partner for that subject) true abstinence (periodic abstinence and coitus interruptus are not acceptable methods of contraception) only female sexual partners

*hCG* human chorionic gonadotropin, *HL* hearing level, *SPL* sound pressure level

**Table 2:** Exclusion criteria of the PROHEARING clinical trial

	Exclusion criteria
1.	Ossification or any other cochlear anomaly that might prevent complete insertion of the electrode array, as confirmed by medical examination and tests including imaging (e.g., digital volume tomography, DVT)
2.	Signs of retrocochlear or central origin to hearing impairment as confirmed by medical examination and tests including imaging (e.g., DVT)
3.	Medical or psychological conditions which contraindicate surgery (e.g., active middle ear infections, tympanic membrane perforation)
4.	Pregnancy or lactation
5.	Additional handicaps that would prevent participation in evaluations
6.	Contraindications for ACEMg: hepatopathy (transaminases or γ-GT ≥2 x upper limit of normal, ULN) severe renal insufficiency (serum creatinine >2 x ULN) disposition to kidney stones iron-storage disease (thalassemia, hemochromatosis, sideroblastic anemia) co-medication with vitamin K antagonists heavy smoking (20 cigarettes per day or more)
7	Current participation in any other clinical trial and/or participation in another clinical trial within 30 days before the study begins

*ACEMg* vitamins A, C, E and magnesium, *DVT* digital volume tomography, *ULN* upper limit of normal

### Blinding and randomization

This is a double-blind clinical trial. Patients fulfilling inclusion/exclusion criteria and giving written consent to participate in this study are randomized 1:1 to ACEMg or placebo. The randomization is stratified by the length of the electrodes (short (16 and 20 mm) versus medium (24 mm) versus long (28 mm)) as planned preoperatively (albeit the length of the electrode is finally determined during surgery). Randomization is performed centrally using a web-based randomization tool. Randomization lists are used to prepare blinded study treatments. The manufacturer provides optically identical investigational medicinal products (IMP) of ACEMg and placebo. Sealed envelopes for emergency unblinding were prepared prior to the study and handed out to the investigator. In addition to blinding patients and investigators, the trial statisticians will stay blinded until the blind data review is successfully completed.

### Clinical outcome measures

The primary outcome of the PROHEARING trial will be the hearing loss at the implanted ear at 500 Hz 3 months post FF (hearing loss = 3 month post-FF threshold minus 1–2 days preoperative threshold) measured by air-conducted pure-tone audiometry. Additionally, secondary endpoints are:

1. Hearing loss at the implanted ear measured by pure-tone audiometry at 500 Hz 6, 9 and 12 months post FF
2. Hearing loss measured by pure-tone audiometry for other frequencies (125 and 250 Hz, 1, 2 and 8 kHz) over time (months 3, 6, 9 and 12 post FF)
3. Speech perception by the Oldenburger Satztest (OLSA), month 12
4. Functional Hearing questionnaire NCIQ, months 3 and 12 after FF
5. Impedances (Ω) at all timepoints
6. Occurrence of tinnitus: Tinnitus Questionnaire at all timepoints

Safety endpoints:

1. All serious adverse events (SAEs)
2. All adverse events (AEs) leading to discontinuation of IMP intake

### Investigational medicinal products

#### IMP Soundbites softgel capsules^®^ and Soundbites^®^ drug information

In this study we supply a combination of vitamins A, C and E together with magnesium, called Soundbites^®^ (chewable pills) or Soundbites softgel capsules^®^, to CI patients with residual hearing. The components of Soundbites^®^ and Soundbites softgel capsules^®^ are listed in Table 3.

**Table 3:** List of Soundbites^®^ and Soundbites softgel capsule^®^ components

Component	Milligrams per tablet (label claim)	Milligrams per daily dose (6 tablets per day)	Milligrams per softgel capsule (label claim)	Milligrams per daily dose (2 softgel capsules per day)
β-carotene	3.0	18	9.0	18
Ascorbic acid	83.33	499.98	250.0	500.0
*dl*-α-tocopherol acetate	44.5	267	133.5	267
Magnesium	52.5	315	157.5	315

Due to their limited stability Soundbites^®^ (chewing pills) were used until 30 June 2015; thereafter, the dosage form changed to Soundbites softgel capsules^®^ with identical content of active substances.

The dosage of each of the nutrients was chosen to maximize both safety and efficiency. The dosage of the components are below the upper daily limits (UDL) established by the EFSA (European Food Safety Authority).

### Treatment assignment

The study medication is administered only to patients included in this study following the procedures set out in the study protocol.

All patients who have signed an Informed Consent Document are identified by a five-digit patient ID (i.e., 01-001) that is generated by the electronic data capture system and is used to identify the subject throughout the study. The database stores only pseudonymized study data. A particular web-based random tool is implemented for this study into the environment of an electronic data capture system. Each member of the trial staff is identified with a qualified account. The investigator supplies basic demographic and clinical details of the patient and the patient is then allocated to one of the treatments according to the stratified randomization list. The investigator is informed of the blinded kit number of the medication for the respective patient. Allocation is split into two batches: a short-term kit for the preoperative treatment phase (medication for 14 days) and a long-term kit for postoperative treatment (medication for 91 days). All patients are only randomized once, but kit numbers are generated on the day of inclusion for the short-term kit (e.g., labels 0001, 0002, 0003, etc.) and generally 1 day before surgery for the long-term kit (e.g., labels 1001, 1002, 1003, etc.).

Patients withdrawn from the study retain their patient ID, if already given. New patients are always allotted a new patient ID. The randomization schedule is stored at the Institute of Biostatistics at MHH.

### Emergency identification of study medication

In the Institute of Biostatistics only the randomization representative and the medical documentalists have access to the randomization lists and the unblinding information. The trial statisticians will stay blinded until the blind data review is successfully completed.

Emergency envelopes are prepared by the Institute of Biostatistics and handed out to the investigator. If a patient is in potential danger and the investigator needs to know which treatment the patient has received, the patient’s emergency envelope may be opened. This must be documented and justified in the electronic Case Report Form (eCRF) and in the subject’s medical records. Treatment with the study medication must be stopped and all relevant study staff members are informed.

In case of an unblinding requested by the clinical pharmacologist, the member of the pharmacovigilance service will contact one unblinded person responsible for the randomization at the Institute of Biostatistics via telephone and specify the ID of the patient to be unblinded. The Institute of Biostatistics will send an email to the clinical pharmacologist with the relevant unblinding information within two workdays.

#### Dosage/therapy schedule

**Fig. 1:**
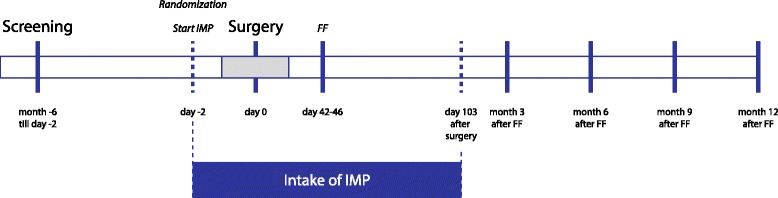
Timeline of the clinical trial PROHEARING. The investigational medicinal product (IMP; ACEMg or placebo) intake starts 2 days before cochlear implant (CI) surgery and lasts for 103 days after surgery. On days 42 to 46 after surgery the first fitting (FF) takes place. At months 3, 6, 9 and 12 after FF study visits are performed

All patients in the study receive treatment with ACEMg or placebo for a total of 106 days. Treatment start is on day 2 preoperatively. The study medication is also taken at the day of surgery and after surgery it is taken for a further 103 days (Fig. 1). Medication is administered twice daily.

#### Treatment plan

Based on strict inclusion and exclusion criteria eligible patients for this trial are identified out of the CI candidates routinely screened in the MHH Clinic of Otolaryngology. Informed consent is obtained from all patients before protocol-specific treatments are carried out. After preoperative audiological functional testing, eligible patients take the IMP per os, starting 2 days prior to CI surgery and continuing for 103 days following surgery. The follow-up lasts for approximately twelve additional months post FF.

A short overview of the treatment and follow-up is given in Table 4.

**Table 4:** Overview of study visits, treatment and follow-up

Study day	Procedure	What is done?	Routine	Study
Between 6 months and 2 days preop	CI screening Including:			
	Hearing tests	Air-conducted and bone-conducted threshold, speech test best aided (with hearing aids) and unaided	x	
		Study audiologist evaluates if audiological inclusion criteria for study are fulfilled Informing the senior physician		x
	Medical talk	Results are discussed	x	
		Possibilities are explained: hearing preservation/conventional electrodes	x	
		Decision about hearing preservation electrode	x	
		Identification for the study Passes potential patients to: assistant physician/study nurse/study audiologist		x
		Information about the CI technique	x	
		Introductory meeting and, when indicated, recruitment (hand out Informed Consent Document)		x
	Following	In critical cases the patient’s documents are shown to the chief physician	x	
		Cost assurance must be checked	x	
		If CI surgery is indicated, the surgery appointment will be made		
	Imaging (e.g., DVT)		x	
Between screening and 2 days preop	Study approval			
		Patient agrees to Informed Consent Document		x
		Study audiologist will be informed		x
	Blood sample	For pregnancy test, if necessary		x
	Blood sample	For γ-GT, transaminases, creatinine		x
	Blood sample	Vitamin E baseline detection		x
	Questionnaire	The patients complete the NCIQ		x
	Imaging (e.g., DVT)		x	
		Pre-decision of electrode length (for study patients FLEX-electrodes are fixed)	x	
Between screening and 3 days preop	Operation appointment			
		The appointment for operation is sent to the patient	x	
Approximately 5 days preop	Patient demonstration			
		All planned surgeries of the coming week are presented	x	
		Overview of the planned study patients, if necessary participating at the discussion		x
Between 10 and 2 days preop	Randomization			
		Fill out patient inclusion form in web-randomization tool Inclusion of the patient in the eCRF database		x
		Inform the HCTC about patient’s inclusion in the study		x
2 days preop	Treatment start			
	Treatment	Starting taking IMPs		x
1–2 days preop	Prefinal diagnostics and patient information			
	Patient reception		x	
	Hearing tests	Air-conducted and bone-conducted threshold, speech test best aided (with hearing aids, if available)	x	
	Vestibularis, BERA, PT		x	
	Information about the surgery		x	
Day 0	Cochlear implant surgery			
Between 1 day preop and day 0	Blood sample	Vitamin E blood level		x
	Surgery	The patient has a FLEX-electrode implanted	x	
1–5 days postop	Hearing tests			
	Hearing tests	Air-conducted (if possible) and bone-conducted threshold	x	
	Test tone	Technical check of the implant	x	
		Patients return packing of IMPs taken		x
		Appointment for the first activation	x	
Usually 4 weeks postop, depending on wound healing	First Fitting			
	Hearing tests	Air-conducted (if possible) and bone-conducted threshold	x	
		Speech tests in quiet and in noise	x	x
	Fitting	Technical check of the implant	x	
	Blood sample	Vitamin E blood level		x
	Questionnaire	The patients complete the NCIQ		x
		Patients return packing of IMPs taken		x
		Appointment for the 3 months post FF follow-up	x	
3, 6, 9 and 12 months post-FF (±14 days each)	Follow-up			
		Patients return packing of IMPs taken		x
	Hearing tests	Speech tests in quiet and in noise	x	x
		Air-conducted and bone-conducted threshold, (aided threshold)	x	
	Fitting	Technical check of the implant	x	
103 days postop		End of IMP intake		x
Only month 6 post FF	Blood sample	Vitamin E blood level		x
Only 3 and 12 months post FF	Questionnaire	The patients complete the NCIQ		x

*CI* cochlear implant, *eCRF* electronic Case Report Form, *DVT* digital volume tomography, *FF* first fitting, *HCTC* Hannover Clinical Trial Center, *IMP* investigational medicinal product, *NCIQ* Nijmegen Cochlear Implant Questionnaire, *postop* posoperatively, *preop* preoperatively

### Study procedures

All study procedures and their relevant performance dates are listed in the study calendar (Table 5).

**Table 5:** Study calendar

		Study approval	ACEMg treatment								
Period/visit	CI screening	Baseline		Baseline audiometry	Surgery						
Point of time	Between −6 months and −3 days		−2 days	−(1–2) days	Day 0	Days 1–5	First fitting (FF) Days 42–46 (±14 days)^d^	Month 3 after FF (±14 days)	Month 6 after FF (±14 days)	Month 9 after FF (±14 days)	Month 12 after FF (±14 days)
Assessment											
Inclusion/exclusion criteria	x										
Demographic data (subject identification)	x										
Medical history	x										
Concurrent medications	x			x		x	x	x	x	x	x
Imaging (e.g., DVT)		x									
Anticipated electrode length		x									
Adverse event assessment^a^			x	x	x	x	x	x	x		
Physical examination^b^	x										
Aetiology of hearing loss	x										
Informed consent		x									
Blood sample (ACEMg)		x			x		x		x		
Questionnaire (NCIQ)		x					x	x			x
Tinnitus Questionnaire		x		x		x	x	x	x	x	x
Pregnancy test		x									
Blood sample for γ-GT, transaminases, creatinine		x									
IMP intake^c^			x	x	x	x	x	x			
Number of returned IMP						x	x	x	x	x	x
Implantation date					x						
Surgeon					x						
Implanted electrode length					x						
Audio processor activation							x				
Technical check of the implant (impedances)					x	x	x	x	x	x	x
Audio processor fitting							x	x	x	x	x
Audiometric test: air and bone conduction (unaided condition)	x			x		x	x	x	x	x	x
Audiometric test: warble-tone air conduction in free field ES/AS (if possible)							x	x			
Speech test: OLSA best aided (like) preoperatively				x							
Speech test: ES only, AS only (if available), ES + residual hearing or EAS (if available) condition							x	x	x	x	x

^a^The adverse event documentation period for this trial begins upon first administration of the IMP(s) and ends 30 days after the last application of the investigational medical product. In case of continuation of any adverse event the documentation period will be prolonged until all adverse events are resolved or until the investigator assess the adverse events as “chronic” or “stable”

^b^The physical examination includes: medical history, audiometry, otoscopy, imaging (e.g., DVT)

^c^The IMP will be taken 2 days preoperatively until 103 days postoperatively

^d^This timepoint takes 5 days (from Monday to Friday). All assessments are made only once

*ACEMg* vitamins A, C, E and magnesium, *AS* acoustic stimulation, *DVT* digital volume tomography, *EAS* electric acoustic stimulation, *ES* electric stimulation, *IMP* investigational medicinal product, *NCIQ* Nijmegen Cochlear Implant Questionnaire, *OLSA* Oldenburger Satztest

#### Recruitment and screening

The MHH Clinic of Otolaryngology is one of the world’s largest CI centers. Patients suffering from hearing loss are transferred to our clinic for further diagnostics and therapy. Based on the information collected during routine CI preliminary investigation potential candidates for the study are asked if they are interested in this study by a study staff member (approved investigator for recruitment as documented in the delegation log). CI screening includes a medical examination, pure-tone audiogram and the Freiburg Speech Test at 65 dB best aided to test the speech intelligibility. Air-conducted hearing thresholds at the ear to be implanted have to be less or equal to 85 dB HL at 125, 90 dB HL at 250 Hz and better or equal than 95 dB HL at 500 Hz. The auditory speech understanding must be less or equal to 60 %. Before inclusion in this clinical trial, patients have to answer additional questions about their health and lifestyle. The inclusion of patients in this clinical trial depends on the results of these investigations. Only if the patient fulfills all inclusion and exclusion criteria and agrees to sign the Informed Consent Document is he recruited for the study. A quiet office is used for these face-to-face private interviews. Approved medical doctors will be conducting introductory meetings with potential subjects.

#### Audiological tests

All audiological testing is performed in an acoustic insulated chamber. An overview of the tests to be performed is given in Table 6. Additionally, the list is plotting, on which study visit which test is performed. Details are explained in the following section.

**Table 6:** Audiological tests per study visit. This table plots on which study visit a particular test is performed

Test	Condition	1–2 days preop	1–4 days postop	4 weeks postop	3, 6, 9 and 12 months post FF
Pure-tone, air-conduct	Unaided	X	X	X	X
Pure-tone, air-conduct	Unaided-contralateral	X		X	X
Pure-tone, bone-conduct	Unaided	X	X	X	X
Pure-tone, bone-conduct	Unaided-contralateral	X		X	X
OLSA training		X		X	X
OLSA in noise	Best aided (like) preop in implantable ear	X^a^			
OLSA in noise	EAS or ES + residual hearing			X	X
OLSA in noise	ES and ipsilateral closed ear canal			X	X
OLSA in noise	AS if activated			X	X
Warble	ES			X	X^b^
Warble	AS if available			X	X^b^

*AS* acoustic stimulation, *EAS* electric acoustic stimulation, *ES* electric stimulation, *OLSA* Oldenburger Satztest

^a^Only if hearing aids are available

^b^Only 3 months post first fitting

##### Pure-tone audiometry

Pure-tone audiometry is performed using a calibrated audiometer according to DIN EN 60318 to detect the acoustic hearing threshold in both ears: the one to be implanted and the contralateral one.

*The test method follows DIN ISO 8253 with headphones for air conduction and a headset for bone conduction in both ears.*

##### Warble

The warble-tone audiometry is the audiometrically determined audiogram of patients supplied with the device (processor) by single tones in free field. It is measured with the same audiometer as pure-tone audiometry and gives feedback about the fitting of the processor for the CI engineer. The warble tones are frequency-modulated pure tones which are used to avoid standing waves. Electric stimulation (ES) and acoustic stimulation (AS) are measured separately to detect the individual hearing thresholds with each component of the processor.

##### Freiburg Speech Test

The most commonly used speech perception test is K.H. Hahlbrock’s word test (1953) described as the Freiburg Speech Test (Freiburg Numbers and Word Test). Hearing loss is declared in percent for words and decibels for numbers. The test is presented without additional noise. The Freiburg Speech Test consists of number words, which are double-digit and mostly four-syllable (10 groups of 10 numbers) and monosyllabic words (20 groups of 20 words).

##### Oldenburger Satztest (OLSA)

This is an adaptive sentence test in noise [10] and shall be performed with a fixed noise level and a variable signal (speech) level to reflect a real-life situation. This test establishes the speech reception threshold in noise. The speech reception threshold is defined as 50 % speech reception in noise and calculated by counting the number of correctly understood words.

#### Impedance measurements

To determine the electrode functionality impedance measurements are performed using the appropriate measurement system of the relevant implant developer.

#### Blood samples

Blood is sampled between screening and 2 days preoperatively to exclude contraindications and to determine vitamin E baseline levels. Additional blood samples are taken 1 day preoperatively or at the day of implantation, at the FF visit 1 month after surgery and 6 months after the FF.

##### Serum levels for γ-GT, transaminases and creatinine

To exclude hepatopathy and severe renal insufficiency as contraindications for ACEMg (see exclusion criteria), blood samples for γ-GT, transaminases and creatinine are taken at the screening visit.

##### Serum pregnancy test

If there is no negative pregnancy test submitted (for women aged between 18 and 50 years; not necessary for women older than 50 years who are at least 1 year menopausal) blood samples for pregnancy tests are taken between the screening appointment and treatment start (2 days preoperatively) if necessary. The pregnancy test is accomplished by means of “HCG STAT” instruction (Roche) and has been standardized against the 4th International Standard for Chorionic Gonadotropin from the National Institute for Biological Standards and Control (NIBSC) code 75/589.

##### Serum vitamin E level

For ACEMg blood level detection only vitamin E (*dl*-α-tocopherol) is determined as a parameter to correlate vitamin serum levels with the hearing loss during the final data analysis. Blood samples for ACEMg blood level detection are taken by standard venipuncture at various timepoints (Table 4).

For the measurement of vitamin E a photometric detection is performed with the aid of a high-performance liquid chromatography (HPLC) Complete Kit (Recipe®).

#### Questionnaire

The Nijmegen Cochlear Implant Questionnaire (NCIQ), a quantifiable, self-assessment health-related quality of life (QoL) instrument for use in CI users is used to evaluate the subjective scoring of QoL. In this test three principal domains are distinguished: physical, psychological and social.

### Pharmacovigilance

The EU Clinical Trials Directive 2001/20 provides the definitions of adverse events (AEs) and reactions used in this trial. Because the IMP is a composition of vitamins and magnesium, it is expected that only few of the participants will experience AEs. All AEs will be primarily documented in the patient’s source documents. In addition to the documentation in the source documents, AEs will be documented in the eCRF in the following cases:

- An AE is serious
- An AE leads to discontinuation of IMP intake

The AE documentation period for this trial begins upon first administration of the IMP(s) and ends 30 days after the last application of the IMP. In case of continuation of any AE the documentation period will be prolonged until all AEs are resolved or until the investigator assess the AEs as “chronic” or “stable.”

Documentation of AEs must be performed in a timely manner on the respective AE Forms in the patient folder and in the eCRF.

An adverse reaction is an AE which is related to the administration of the study drugs. If any adverse reactions arise, they will be reported on the AE Form within the CRF. If a serious adverse event (SAE) thought to be related with the study drug occurs, reporting will follow the regulatory requirements and will be reported to the competent authority and the sponsor within 24 hours of our becoming aware of the event.

All suspected unexpected serious adverse reactions (SUSARs) will be the subject of expedited reporting. Fatal or life-threatening SUSARs must be reported within 7 days, all other SUSARs need to be reported within 15 days.

Any pregnancy occurring within the reporting period as well as significant overdosing must be reported as a SAE.

### Sample size calculation

Sample size is feasibility driven as cochlear implantation with residual hearing is a relatively rare condition. Initial sample size estimation was based on historical data from the MHH Department of Otolaryngology with patients receiving CI electrodes of different lengths. The type-I error was set at 5 % (two-sided) and sample size was calculated for a power of 80 % using a two-group *t* test. A change in hearing thresholds from baseline to 3 months after FF of 15.6 dB with a standard deviation of 15.5 dB at 500 Hz has been observed. For the sample size calculation it has been assumed that the standard deviation is the same in the placebo and in the active treatment group and that the mean change in hearing thresholds is 5 dB smaller with ACEMg therapy compared to placebo. Under these assumptions a required sample size of 150 patients per group was targeted. However, recruitment has been difficult up to now and a total of 300 patients cannot be enrolled in a reasonable time frame. The sample size target was reduced to 70 patients per group in a recent amendment. The power to detect a relevant 5-dB treatment effect decreases to approximately 50 % (which means an increase in the type-II error to 50 %), but a total of 140 patients should be sufficient to decide in this phase II trial whether ACEMg offers a promising treatment concept for protection of residual hearing in CI. The power increases to 80 % if the treatment difference is in the order of 7.5 dB. No interim analysis will be performed.

### Statistical analysis

The primary analysis is based on the intention-to-treat (ITT) population consisting of all randomized patients who took the study medication at least once. The primary null hypothesis of equal hearing loss in ACEMg and placebo will be evaluated with an analysis of covariance (ANCOVA) model including the factor treatment (ACEMg versus placebo), the baseline residual hearing, surgeon, planned length of electrode and formulation of medication (chewing pills versus softgel capsules). The null hypothesis, of no smaller reduction in residual hearing for ACEMg patients, is rejected if the upper bound of the ANCOVA-derived two-sided 95 % confidence interval of the difference in change from baseline (ACEMg minus placebo) is less than 0.

To evaluate the robustness of the estimated treatment effect, the primary analysis will be repeated based on the per-protocol population. In addition, the primary objective is evaluated with the nonparametric, stratified Gehan test as a sensitivity analysis. Moreover, analysis will be conducted using real length of electrode instead of planned length of electrode as independent factor.

Secondary analyses will be conducted on the ITT population and on the per-protocol population as sensitivity analyses. Key secondary analyses include the comparison of ACEMg group to placebo in terms of residual hearing for different frequencies and different timepoints after surgery. All analyses will be performed in line with the primary analysis using ANCOVA. Differences in the treatment effects within patient groups with different electrode length are evaluated descriptively.

Absolute and relative frequencies of (serious) adverse events will be listed for each treatment group separately. Differences will be analyzed in a descriptive manner. The safety population will be used for analysis.

#### Extent of study treatment exposure and compliance

To quantify compliance to the study medication, serum vitamin E levels are measured in all randomized patients before, during (at surgery and 4 weeks postoperatively) and after treatment period. Moreover, patients return their treatment kits to the study site and provide information on their compliance via a short, self-administered questionnaire.

Patients under ACEMg treatment are only classified as compliant (and used for per-protocol analysis):

- If their vitamin E level 4 weeks postoperatively is ≥20 μg/ml

Nevertheless an ACEMg patient will be classified as noncompliant even though their vitamin E level is ≥20 μg/m if:

- The patient states in the questionnaire a noncompliance >50 %, and/or
- The returned treatment kits contains >50 % of the study medication, whereas missing bottles or blisters are considered as full bottles or blisters

Patients in the placebo group with postoperative vitamin E levels ≥20 μg/ml are most likely to be noncompliant to the study procedures, since they agreed in their informed consent not to use daily multivitamins or other supplements during the course of the study, but show vitamin E levels that are unrealistic without vitamin supplements.

Patients under placebo are classified as noncompliant:

- If their vitamin E level 4 weeks postoperatively is ≥20 μg/ml, and/or
- If the patient states in the questionnaire a noncompliance >50 %, and/or
- If the returned treatment kits contain >50 % of the study medication, whereas missing bottles or blisters are considered as full bottles or blisters

### Data handling conventions and missing values

The primary endpoint is residual hearing measured in decibels for a frequency of 500 Hz by air-conducted pure-tone audiometry at the implanted ear. If the patient reaches the upper detection limit of the 500-Hz air-conducted pure-tone audiometry of 110 dB without hearing, the measurement is set to 120 dB as the upper limit of detection + 10 dB. This “upper limit +10” approach is used in all analyses concerning residual hearing (for all frequencies).

The anticipated drop-out rate is expected to be low in PROHEARING as patients in the initial phase after CI are closely followed for clinical reasons. Frequency and reasons for drop-out will be recorded as far as possible and will be analyzed in both study arms. Missing values in the ITT analysis of pure-tone audiometry are replaced by worst possible value, that means that a missing value at any follow-up visit is replaced by the respective upper limit of quantification + 10 dB.

### Data management

All study data are collected by study personnel. Data are entered via an eCRF in a clinical trial database. For each patient enrolled an eCRF must be completed after the patient’s visit. This also applies to records for those patients who fail to complete the study. Only SAEs will be additionally documented and notified on paper forms.

Verification of the data in the eCRF occurs by monitoring as well as via range, validity, and consistency checks programmed in the system. In certain cases, queries can be detected by the study software or by an authorized study staff member. Based on the queries, the investigator can review and answer the discrepancies that are found directly in the system. All changes of data entered in the eCRF can be followed by an audit trail.

A quality control will be performed before the database is closed. This procedure is documented. After the close-out visit at the study center the access of the investigator to the eCRF/database is changed to a “read-only” access (modification of data is no longer possible). After database closure the investigator and the data manager can only read the data, they can not change them; the final data set is transferred to the Institute for Biometry for statistical analysis of the SAS data.

### Patient information/informed consent and data protection

The investigator is responsible for obtaining the patient’s written informed consent after adequate explanation of the aim, study assessments, potential risks, benefits, and consequences of the study, as well as alternative treatment options. The patient information/informed consent form has to be signed in duplicate by the patient and the investigator. One document will be given to the patient, the other remains in the Trial Investigator File (TIF) at the trial site. No study procedures are allowed to be conducted until the patient’s written informed consent has been obtained.

The patient information/informed consent form has to be revised whenever important new information becomes available that may be relevant to the subject’s consent. The patients have to be informed and asked to give their consent to continue study participation by signing the updated form.

All study staff members have to give due consideration to data protection and medical confidentiality. The collection, transfer, storage and analysis of personal study-related data are performed pseudonymized according to national regulations. The declaration of data protection is contained within the patient information/informed consent form.

### Monitoring and audits/inspections

To ensure compliance with the protocol, to legal and regulatory requirements applicable for clinical trials and with the ethical principles of the Declaration of Helsinki and the International Conference on Harmonization of Technical Requirements for Registration of Pharmaceuticals for Human Use (ICH) and GCP, monitoring visits are scheduled to take place during the study. At those regular monitoring visits, the monitor reviews the eCRF for completeness and clarity and performs source data verification. Source data are defined as any printed, optical and electronic document containing source data (e.g., hospital records, laboratory notes, drug accountability records). The monitor also reviews notification of SAEs and the TIF.

Audits (by the sponsor) and inspections (by regulatory authorities) may be performed in order to verify that the clinical study is performed according to the study protocol as well as to other applicable regulatory requirements. The auditor or inspector is independent in regard to personnel involved in the conduct of this clinical trial. This may occur at any time from start to after closure of the study.

Generally the sponsor quality manager audits the conduct of a clinical trial on a regular basis (determination of frequency is risk-adapted; usually annually). Inspections by regulatory authorities happen infrequently and cannot be foreseen by the investigator or sponsor.

The establishment of a data monitoring committee (DMC) for this clinical study was not considered necessary by the sponsor as a “non-critical indication where patients are treated for a relatively short time and the drugs under investigation are well characterized and known for not harming patients” (cited from “Guideline on data monitoring committees,” EMA 27 July 2005) is being investigated. In addition, safety endpoints are verified by the monitor during periodic monitoring visits and reported to the sponsor within the written report after each visit.

## Discussion

The insertion of a CI into the inner ear is to some extent potentially damaging to the inner ear tissue, which can affect the endocochlear potential, create oxidative stress, and initiate proapoptotic pathways associated with direct injury to, and loss of, hair cells [11]. Even though to our knowledge the exact mechanisms involved during CI surgery that results in loss of residual hearing are not completely understood recent findings on caspase activation, JNK activation, oxidative stress with reactive oxygen species, and lipid peroxidation of cellular membranes [12] give a first hint of possible targets for drug-based therapies of insertion trauma-related hearing loss.

There is an increasing role for antioxidants in the protection of residual hearing loss as there is more evidence that links high levels of oxidative stress to programmed cell death of the remaining hair cells. Knowledge of the mechanisms underlying noise-induced hearing loss (NIHL) and drug-induced hearing loss (DIHL) has led to the hypothesis that cochlear-implantation-related hearing loss is based on the same pathophysiology. Noise and ototoxic drugs induce free radical formation in the inner ear, which up-regulate apoptotic cell-death genes [13]. If free radical build up is sufficient, cell membranes are attacked, resulting in necrotic cell death [13].

Some exogenous antioxidants were tested in animal models and some degree of benefit has being shown against cisplatin, carboplatin, aminoglycoside, and noise-induced trauma to the inner ear: D-methionine (D-met [14]); ascorbic acid (vitamin C [15]); *dl*-α-tocopherol (vitamin E [16]); *N*-acetylcysteine (NAC [17, 18]). Clinical trials examining the benefit of antioxidants on hearing loss of different origins are various and include NAC (ClinicalTrials.gov Identifier: NCT00552786), NAC + Mg (ClinicalTrials.gov Identifier: NCT01727492) or ACEMg (ClinicalTrials.gov Identifier: NCT00808470), which has been evaluated for the treatment of NIHL. NAC (ClinicalTrials.gov Identifier: NCT02094625) has been tested for the prevention of cisplatin-induced hearing loss or sodium thiosulfate to prevent carboplatin- [19] or cisplatin-induced hearing loss (ClinicalTrials.gov Identifier: NCT01369641; ClinicalTrials.gov Identifier: NCT00716976; ClinicalTrials.gov Identifier: NCT02281006). Prevention of drug-induced ototoxicity is being tested by NAC treatment (ClinicalTrials.gov Identifier: NCT01131468). But, to our knowledge, only one study has addressed hearing loss associated with electrode insertion trauma and cochlear implantation. In this study the researchers were able to demonstrate benefit with NAC treatment [17]. This benefit only pertained to the high frequencies of the basal turn and did not extend significantly to low-frequency hearing located in the apical section of the cochlea [17]. Unfortunately, delivery of NAC to the round window prior to implantation caused a slight increase in hearing thresholds and greater amounts of osteoneogenesis, which may preclude its use locally in the protection of residual hearing [17]. However, there have been no randomized controlled trials to investigate antioxidant effects on CI electrode-insertion-related hearing loss. The study protocol of this clinical trial allows controlling of all parameters influencing residual hearing after cochlear implantation. Performing all surgeries and patient follow-up at one study side leads to maximum consistency in diagnostics and therapy by there being less variability in surgery and audiological testing techniques and fitting. This approach will allow a better detection of the influence of ACEMg on residual hearing in CI patients.

The dosage of vitamin C (magnesium ascorbate; 500 mg), magnesium (magnesium citrate, magnesium ascorbate, magnesium stearate; 315 mg), vitamin E (*dl*-α-tocopherol acetate; 267 mg), and β-carotene (18 mg) administered in this placebo-controlled trial was chosen to find the correct balance between a potential therapeutic effect and the suggested tolerable upper intake level: the maximum level of total chronic daily intake of a nutrient (from all sources) judged to be unlikely to pose a risk of adverse health effects to humans [20].

If the hypothesis that decreasing free radicals with this formulation of micronutrients will preserve residual hearing in CI patients is correct, then these patients will benefit from better hearing sensation.

### Trial status

The PROHEARING trial is ongoing, with 51 patients recruited as of June 2015. The last patient out is expected to be in July 2018.

## Abbreviations

ACEMg, vitamins A, C, E and Magnesium; AE, adverse event; BfArM, Bundesinstitut für Arzneimittel und Medizinprodukte; CI, cochlear implant; DIHL, drug-induced hearing loss; DVT, digital volume tomography; EAS, electric acoustic stimulation; eCRF, electronic Case Report Form; EFSA, European Food Safety Authority; FF, first fitting; GCP, Good Clinical Practice; GMP, Good Manufacturing Practice; hCG, human chorionic gonadotropin; HCTC, Hannover Clinical Trial Center; HL, hearing level; IMP, investigational medicinal product; ITT, intention-to-treat; MHH, Hannover Medical School; NCIQ, Nijmegen Cochlear Implant Questionnaire; NIHL, noise-induced hearing loss; QoL, quality of life; SAE, serious adverse event; SPL, sound pressure level; SUSAR, suspected unexpected serious adverse reaction; UDL, upper daily limits; ULN, upper limit of normal

## References

[1] Lenarz T, Stover T, Buechner A, Lesinski-Schiedat A, Patrick J, Pesch J (2009). Hearing conservation surgery using the Hybrid-L electrode. Results from the first clinical trial at the Medical University of Hannover. Audiol Neurootol.

[2] Lenarz T, James C, Cuda D, Fitzgerald O'Connor A, Frachet B, Frijns JH (2013). European multi-centre study of the Nucleus Hybrid L24 cochlear implant. Int J Audiol.

[3] Talbot KN, Hartley DE (2008). Combined electro-acoustic stimulation: a beneficial union?. Clin Otolaryngol.

[4] Skarzynski H, Lorens A, Zgoda M, Piotrowska A, Skarzynski PH, Szkielkowska A (2011). Atraumatic round window deep insertion of cochlear electrodes. Acta Otolaryngol.

[5] Helbig S, Baumann U, Hey C, Helbig M (2011). Hearing preservation after complete cochlear coverage in cochlear implantation with the free-fitting FLEXSOFT electrode carrier. Otol Neurotol.

[6] Jurawitz MC, Buchner A, Harpel T, Schussler M, Majdani O, Lesinski-Schiedat A (2014). Hearing preservation outcomes with different cochlear implant electrodes: Nucleus(R) Hybrid-L24 and Nucleus Freedom CI422. Audiol Neurootol.

[7] Le Prell CG, Hughes LF, Miller JM (2007). Free radical scavengers, vitamins A, C, and E, plus magnesium reduces noise trauma. Free Radic Biol Med.

[8] Abi-Hachem RN, Zine A, Van De Water TR (2010). The injured cochlea as a target for inflammatory processes, initiation of cell death pathways and application of related otoprotective strategies. Recent Pat CNS Drug Discov.

[9] Dinh CT, Van De Water TR (2009). Blocking pro-cell-death signal pathways to conserve hearing. Audiol Neurootol.

[10] Wagener K, Brand T, Kollmeier B (1999). Entwicklung und Evaluation eines Satztests für die deutsche Sprache I: Design des Oldenburger Satztests (Development and evaluation of a German Sentence Test part I: Design of the Oldenburg Sentence Test). Z Audiol.

[11] Bas E, Dinh CT, Garnham C, Polak M, Van de Water TR (2012). Conservation of hearing and protection of hair cells in cochlear implant patients with residual hearing. Anat Rec (Hoboken).

[12] Eshraghi AA, Gupta C, Van De Water TR, Bohorquez JE, Garnham C, Bas E (2013). Molecular mechanisms involved in cochlear implantation trauma and the protection of hearing and auditory sensory cells by inhibition of c-Jun-N-terminal kinase signaling. Laryngoscope.

[13] Henderson D, Bielefeld EC, Harris KC, Hu BH (2006). The role of oxidative stress in noise-induced hearing loss. Ear Hear.

[14] Samson J, Wiktorek-Smagur A, Politanski P, Rajkowska E, Pawlaczyk-Luszczynska M, Dudarewicz A (2008). Noise-induced time-dependent changes in oxidative stress in the mouse cochlea and attenuation by D-methionine. Neuroscience.

[15] Derekoy FS, Koken T, Yilmaz D, Kahraman A, Altuntas A (2004). Effects of ascorbic acid on oxidative system and transient evoked otoacoustic emissions in rabbits exposed to noise. Laryngoscope.

[16] Hou F, Wang S, Zhai S, Hu Y, Yang W, He L (2003). Effects of alpha-tocopherol on noise-induced hearing loss in guinea pigs. Hear Res.

[17] Eastwood H, Pinder D, James D, Chang A, Galloway S, Richardson R (2010). Permanent and transient effects of locally delivered n-acetyl cysteine in a guinea pig model of cochlear implantation. Hear Res.

[18] Lu J, Li W, Du X, Ewert DL, West MB, Stewart C (2014). Antioxidants reduce cellular and functional changes induced by intense noise in the inner ear and cochlear nucleus. J Assoc Res Otolaryngol.

[19] Neuwelt EA, Brummett RE, Doolittle ND, Muldoon LL, Kroll RA, Pagel MA (1998). First evidence of otoprotection against carboplatin-induced hearing loss with a two-compartment system in patients with central nervous system malignancy using sodium thiosulfate. J Pharmacol Exp Ther.

[20] EFSA. European Food and Safety Agency: tolerable upper intake levels for vitamins and minerals 2006. Available from: http://www.efsa.europa.eu/sites/default/files/efsa_rep/blobserver_assets/ndatolerableuil.pdf. Accessed 31 Aug 2012.

